# Correction: Dynamics of international Trade: A 30-year analysis of key exporting nations

**DOI:** 10.1371/journal.pone.0334340

**Published:** 2025-10-10

**Authors:** Nobuo Yazawa

In Fig 9, there is an error in the R code used in the clustering analysis. The distance matrix argument should be “d=dist_matrix” not “d=d”. Please see the correct Fig 9 here.

**Fig 9:**
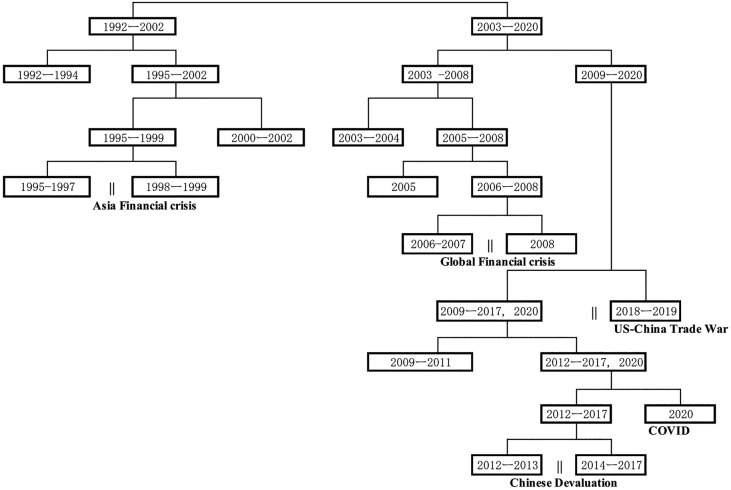
Dendrogam of hierarchical clustering analysis.
