# Supplementary material for: Dynamics of international Trade: A 30-year analysis of key exporting nations
Source: PLoS One. 2023 Aug 1;18(8):e0289040. doi: 10.1371/journal.pone.0289040 (PMC10393138; doi:10.1371/journal.pone.0289040)
Supplement: Attachment — Submitted filename: Response to reviewers.docx [file pone.0289040.s006.docx]

**Response to reviewers**

**Dear Dr. Radulescu and Reviewers,**

We would like to commence by thanking Dr. Radulescu and the reviewers for their valuable time and constructive comments. Their expert knowledge of the field has helped us to strengthen the manuscript significantly. According to the valuable suggestions provided by the reviewers, we have revised the manuscript to the best of our abilities, and revised text to meet PLOS ONE style requirements.. We endeavored to address all the comments and our reflections are given below point by point.

Sincerely,

Nobuo Yazawa

Beppu University

yazawa@nm.beppu-u.ac.jp

Review Comments to the Author

We have highlighted the reviewer's comments in blue, and the additions made to the manuscript are enclosed within ruled lines.

Reviewer #1:

Comments: The authors studied “shifting dynamics in international trade networks” in US, China, India, Japan and South Korea. This paper needs improvement as has been listed below:

*Response: I am profoundly grateful for your invaluable comment, which has significantly enhanced the quality of our paper. I will now systematically address each question in sequence.*

1.Abstract

Reviewer#1’s comment 1: the policy proposal of the study's originality and importance should be written in this section.

*Response: Thank you for your valuable feedback. I have revised the specified section to highlight the policy proposal of the study's originality and importance. The section now includes the following sentences:*

*‘Our findings inform several policy recommendations. These include encouraging balanced trade for economic stability and improved international relations, realigning trade focus in response to the shift in trade network center, and developing resilience policies that account for the substantial impact of global events on trade networks.’*

Reviewer#1’s comment 2: explaining the method and variable in one sentence would be sufficient.

*Response: Response: Thank you for your suggestion. Considering that we utilized three distinct methods, we have tried to succinctly describe our methodology and variables in three sentences:*

*'First, we examined time-series trade data during the study period and quantified network reciprocity through the sum of squared trade imbalances across different product categories. Second, we visualized these trade networks using arrows, with their sizes being proportional to the trade value between each pair of countries; significant trade relationships were indicated by arrows with a standard deviation value of 55 or above. Finally, we introduced a new cluster analysis methodology for studying the evolution of network structures over time. This method utilized an 80-dimensional vector representation of the annual networks, divided into four categories, and the resulting structures were visualized as dendrograms using R software.'*

Reviewer#1’s comment 3: the policy proposal of the study's originality and importance should be written in this section.

*Response: Thank you for your feedback. It appears that this comment is a repetition of Comment 1. As previously responded in my reply to Comment 1, [Our findings inform several policy recommendations. These include encouraging balanced trade for economic stability and improved international relations, realigning trade focus in response to the shift in trade network center, and developing resilience policies that account for the substantial impact of global events on trade networks]. I hope this adequately addresses your concern, and please let me know if there are further questions or points of clarification.*

2.Introduction

Reviewer#1’s comment 4: the main motivation of the study should be explained correctly in this section.

*Response: Thank you for your valuable feedback. I have revised the introduction to clarify the main motivation of the study. The revised text now reads:*

*' The motivation for this study lies in the quest to comprehend international trade from a network perspective. Although international trade has been extensively studied, the network approach remains relatively uncharted territory. We posit that this perspective could provide novel insights into the dynamics and resilience of trade relationships amidst global challenges, emphasizing the interconnectedness of worldwide trade. This study will augment the existing literature by offering a fresh lens to view international trade dynamics.’*

Reviewer#1’s comment 5: the study's importance, purpose and t**heoretical framewo**rk should be discussed in detail.

*Response: Thank you for your insightful feedback. In response, I have expanded this section with the following details:*

*‘Our objective is to examine the evolution in structural properties, including centrality and reciprocity, of trade networks among the United States (U), China (C), India (I), Japan (J), and South Korea (K) from 1992 to 2020. The significance of our work lies in its unique perspective on international trade dynamics, focusing on an underexplored network approach. By investigating the interconnectedness of global trade, we aim to offer novel insights into the resilience of trade relationships amidst global challenges and the fluidity of these dynamics.*

*The theoretical framework of our paper integrates the following components:*

*1.International Trade Theory: This theory serves as the bedrock for comprehending and analyzing international trade dynamics. It is employed in our exploration of evolving trade relationships among the U.S., China, India, Japan, and South Korea over 29 years. The theory aids in interpreting trade imbalances, shifts in trade dominance, and their responses to various political and economic crises.*

*2.Network Analysis: This methodological approach enables us to scrutinize the structure of international trade relationships. Network analysis aids in visualizing and understanding intricate relationships between trade partners, providing insights into the concepts of centrality and reciprocity. We adopt hierarchical clustering as a technique for community detection in network theory.*

*3. Time-Series Analysis: This analytical technique is used to examine data points collected at regular time intervals. In our study, we apply time-series analysis to reveal how trade networks' structure has evolved over time and to assess the impact of significant events, such as political and economic crises.*

*4. Crisis Theory: We use this theory to gauge the impact of political and economic crises on international trade networks. It provides an analysis of how these crises disrupt trade flows, alter trade networks' structure, and shift the centrality and reciprocity of trading partners.*

*Our methodological approach involves gathering trade data between the five countries over the 29-year period, utilizing network analysis tools to interpret the structure and dynamics of their trade relationships. This is supplemented by a time-series analysis to pinpoint trends and patterns over time, and an application of crisis theory to understand the influence of political and economic crises on these trade networks.’*

Reviewer#1’s comment 6: theoretical explanations regarding the main topic is insufficient.

*Response: We appreciate your feedback on the need to provide more detailed discussion of the study's importance, purpose, and theoretical framework, as well as to provide more theoretical explanations regarding the main topic. We understand these two comments are closely related.*

*In response to these comments, we have made considerable revisions to our Introduction section. These revisions include a thorough elaboration of the study's importance and purpose, a comprehensive discussion of the theoretical framework, and a detailed exposition of theories related to the main topic. Specifically, we have delved into the International Trade Theory, Network Analysis, Time-Series Analysis, and Crisis Theory, explaining their relevance to our study and how they inform our research design and analysis.*

*We believe these additions address both comments adequately by giving the reader a more in-depth understanding of the theoretical basis of our study and its significance in the broader context of international trade dynamics. We hope that the revised Introduction section will now meet your expectations.*

Reviewer#1’s comment 7: the general plan of the study should be written at the end of the section.

*Response: Thank you for your valuable suggestion. We agree that including a brief overview of the study's plan at the end of the section would enhance the paper's clarity and flow. Accordingly, we have incorporated a summary of our research plan as follows:*

*‘ By adopting a systematic approach, our study examines the structural characteristics of international trade networks. Leveraging international trade theory, we delve into the dynamics of trade relationships over a 29-year period. Through network analysis, we visualize these relationships, understanding their centrality and reciprocity. Time-series analysis helps us identify trends and changes over time, while crisis theory enlightens us on the effects of significant political and economic crises on these networks. We blend these theoretical perspectives and methodological tools to present a holistic analysis of international trade dynamics.’*

*We hope this summary provides a clear and concise outline of our study's general plan, addressing the concern raised.*

3.Literature

Reviewer#1’s comment 8: it is very important to approach literature studies critically. However, a literature review was not conducted in the study. I suggest creating a literature table.

*Response: Thank you very much for your valuable suggestion. I added the following table as suggested.*

**Literature table**

| Articles | Their method | Their key findings | Our methods | Our key findings |
| --- | --- | --- | --- | --- |
| [13] | An eigenvector centrality of graph theory was applied as a common measure for assessing the importance of nodes in a network | 2007 marked an inflection point at which new winners and losers emerged and a remarkable reversal of leading role took place between the two major economies, the US and China. | Visualization of the time series progression of network structure with arrows whose standard deviation are 55 or greater. | The center of the network has shifted from U to C in 2007 for all products, 2005 for capital goods, 2006 for intermediate goods. No shift for consumer goods and raw materials. |
| [14] | The study of link reciprocity in binary directed networks of global trade  a definition of reciprocity as the correlation coefficient between the entries of the adjacency matrix of a directed graph year: 1948-2000 | Reciprocity of trade network increased from 0.68 (1948) to 0.9 (2000) | The degree of reciprocity within a network measured by the sum of squared trade imbalances between each pair of actors | the (overall increase in squared trade imbalances) decrease of reciprocity for all products and consumer goods, but the (decrease in the squared trade imbalances) increase of reciprocity for capital goods, intermediate goods, and raw materials. |
| [15] | multi-regional input-output data to decompose 186 national economies into 26 industry sectors 1990-2010  (a) a definition of reciprocity as  $r=\frac{1}{\left\vert A \right\vert}Tr{[A]}^{2}$ where A is an adjacency matrix | (a) The reciprocity (r) gradually increases in the national partition Cc, but saturating in 2000. |  |  |
|  | (b) measure the Hamming distance between the international trade network in the present and the preceding year | (b) Hm is an applicable measure to identify anomalies in trade patterns, such as the financial crisis in 2009 | Clustering analysis applying to time vectors | That the half of the substantial alterations in the network structure occur during such incidents suggests that global trade is significantly susceptible to these crises. |

Reviewer#1’s comment 9: the difference of the study from the literature and its contribution to the literature should be explained under this title

*Response: Thank you for your valuable feedback. I have added the following sentences to this section.*

*‘Our study sets itself apart from existing literature through its unique methodological approach and the ensuing findings. Garlaschelli and Loffredo (2004) [10] centered their research on link reciprocity in binary directed networks, employing the correlation coefficient between adjacency matrix entries of a directed graph to formulate a new reciprocity definition (table 1). We too analyzed reciprocity, but assessed it in a weighted directed network, gauged by the sum of squared trade imbalances between actor pairs. This method simplifies reciprocity measurement compared to matrix computation methods.*

*In the study "A Network of Networks Perspective on Global Trade" (2015) [11], researchers dissected 186 national economies into 26 industry sectors using multi-regional input-output data (table 1). They detected anomalies in trade patterns using measures like the Hamming distance between the International Trade Network (ITN) in the current and preceding year. Contrarily, our research applies clustering analysis to time vectors. We adopted this method because it was innovative to use clustering analysis, commonly used for grouping points distributed in space, to group points distributed over time.*

*The 2022 paper "The rise and fall of countries in the global value chains" [12] employed graph theory's eigenvector centrality to gauge node importance in a network, noting a significant role reversal between the US and China in 2007 (table 1). Our research, however, visualizes time series progression of network structure, observing the network center shifting from U to C in 2007 for all products, in 2005 for capital goods, and in 2006 for intermediate goods. This method's selection is due to visualization's intrinsic ability to communicate simply and clearly, compared to complex methods involving eigenvalue matrix computations.*

*Our research makes distinct contributions to the literature. Firstly, our focus on network reciprocity, measured by the sum of squared trade imbalances, offers a fresh lens to scrutinize the global trade network. This new method helps discern reciprocity trends among various product categories. Secondly, visualizing the time series progression of network structure provides insights into the shifting network center, complementing the eigenvector centrality measure used in previous studies. Lastly, applying clustering analysis to time vectors introduces a novel method to study alterations in the global trade network. This innovative approach indicates that half of the significant changes in network structure occur during considerable crises, highlighting global trade's vulnerability to such events. Overall, our study enriches the current comprehension of global trade networks by introducing new methodologies and offering fresh insights into their structure and dynamics.*

*Our study contributes to the literature in a few distinct ways. Firstly, our focus on the degree of reciprocity within a network measured by the sum of squared trade imbalances provides a new perspective to examine the global trade network. This novel method helps identify a divergence in reciprocity trends among different product categories. Secondly, the visualization of the time series progression of network structure offers insights into the shifting center of the network. This is a unique angle that complements the eigenvector centrality measure used in prior studies.Thirdly, our use of clustering analysis applying to time vectors introduces a new method of studying changes in the global trade network. This innovative approach suggests that half of the substantial alterations in the network structure occur during significant crises, pointing to the susceptibility of global trade to these events. Overall, our work expands on the current understanding of global trade networks, introducing new methodologies and providing fresh insights into their structure and dynamics.’*

4.Data and Methods

Reviewer#1’s comment 10: analyzes and the findings have not been adequately discussed.

*Response: Thank you for your insightful comment. In response, I have extended the discussion on our analysis and findings in the revised manuscript. This expanded discussion underscores their significance, discusses implications of observed trends, connects the findings to our theoretical framework, compares them with existing research, and explores the influence of major global events on trade imbalances. I believe that these additions and modifications address your comment effectively, thereby enriching the depth and breadth of analysis in the manuscript.*

*‘4.2. Visualization of the time series progression of network structure*

*4.2.6. Total number of links*

*From fig 3, the center of the network has shifted from U to C in 2007 for all products, 2005 for capital goods, 2006 for intermediate goods. There was no shift for consumer goods and raw materials. When we delve into the product category analysis, the study finds differing dominance patterns across product categories and countries over time. For instance, while J was consistently significant in outflows and inflows for all products, U and C fluctuated in dominance over time. This could be due to changing economic landscapes and industrial capacities in these economies. Similarly, for capital goods, we observe the shifting dominance of regions such as U and J to C over the course of the study period. This could potentially reflect industrialization and development trends, particularly in emerging economies such as C. It would be interesting to further investigate how these changes align with socioeconomic transformations in these nations.*

*On the other hand, the consumer goods flow displays a multi-polar network with U, C, and J playing major roles. This could potentially be influenced by factors such as consumer demand, industrial capacity, and trade policies in these regions. The stable nature of this network could suggest an established and resilient trade system. The intermediate goods category exhibits a diverse and increasingly diversified network, possibly reflecting the interconnected nature of global supply chains. This is especially pertinent given the centrality of intermediate goods in the manufacturing process. However, the study also observes a consolidation of hub status in C since 2015, which could indicate a potential vulnerability in the network and warrants further investigation. For raw materials, the study observes U's dominance in outflows and J's dominance in inflows, which could possibly be tied to their respective natural resource endowments, production capacities, and consumption needs. This highlights the critical role of resource allocation in global trade.*

*Overall, it was observed that the network center shifted from U to C in 2007 for all products, in 2005 for capital goods, in 2006 for intermediate goods. The study’s finding that the shift happened in 2007 for all products is in agreement with the previous research [17]. Moreover, our study found that the point at which shifts occurred varies by product category (fig 3). These changes may have broader implications. For example, the shift from U to C as the central node in all product categories (except for consumer goods and raw materials) could reflect broader geopolitical shifts and has potential implications for global economic stability and development. Further, the increase in the number of major arrows for raw materials in 2001, concomitant with 9/11, could suggest an impact of global events on trade patterns. A deeper understanding of these trends and their drivers is crucial to the formation of trade policies.*

*4.3. Hierarchical clustering*

*As indicated in Figure 9, the event of 9/11 is marked as a pivotal point, segregating the entire dataset into two distinct subsets 1992-2000 and 2001-2020. The subset encompassing the years 1992-2000 further divides into two clusters, 1992 and 1993-2000. Then 1993-2000 divides into 1993-1994 and 1995-2000. Moreover 1995-2000 divides into 1995-1997 and 1998-2000, in tandem with the Asian financial crisis. Conversely, the 2001-2020 subset segregates into 2001-2009 and 2010-2020. 2001-2009 bifurcates into 2001-2003 and 2004-2009. 2004-2009 subdivides into 2004-2007 and 2008-2009 clusters, corresponding with the global financial crisis. The 2010-2020 subset bifurcates into two clusters, 2010-2014 and 2015-2020, during the period of China's currency devaluation. In conclusion, the 2015-2020 subset subdivides into the 2015-2017 cluster and the 2018-2020 cluster concurrently with the instigation of the US-China trade war, provoked by Trump’s inauguration. The cluster analysis illuminates that the 9/11 attacks had the most substantial influence on the network structure, succeeded by the global financial crisis. The third considerable impact came from the Asian financial crisis and China's currency devaluation, while the US-China trade war during the Trump administration had the least significant effect on the structure thus far. Of the nine divisions observed in the diagram, five align with crises, suggesting that around half of the significant modifications in network structure occur during incidents like 9/11 and economic crises.*

*Figure 9 demonstrates the alterations in the network structure post various crises, providing crucial insights into the dynamics and resilience of global trade. The occurrence of considerable changes in the network structure during such incidents indicates a substantial vulnerability of global trade to these crises. However, the impact of these crises on the network structure varies.*

*The 9/11 event had the most significant effect, suggesting that global geopolitical events could disrupt trade relationships and reshape trade flows potentially due to political realignments, shifts in regional stability, or interruptions to global supply chains. Additionally, the disruptions post 9/11 were unparalleled, possibly prompting nations to diversify trade links, leading to substantial alterations in the network structure. However, while assessing the 9/11 aftermath, it is crucial to consider that China formally joined the World Trade Organization (WTO) that same year. Disentangling the long-term impacts of 9/11 from those originating from China's WTO accession can be a complex process.*

*In contrast, the US-China trade war under Trump's administration had the least influence on the network structure, indicating that trade disputes, although disruptive, might not significantly impact the global trade network as a whole. This could also suggest adaptability in the network, with other nations compensating for such disputes and thereby preserving the overall structure. The Asian financial crisis, the global financial crisis, and China's currency devaluation corresponded with considerable network structure alterations. These economic crises could induce shifts in regional economic landscapes, affecting trade flows. For instance, a financial crisis might weaken a country's economic stance, curbing its import-export capabilities, and leading to the renegotiation of trade deals.*

*It is essential, however, to note that the correlations identified in this study do not necessarily imply causation, warranting further research to conclusively determine these crises' impact on global trade. In summary, this analysis underscores the dynamic nature of global trade, significantly influenced by major geopolitical and economic crises. Recognizing these dynamics and vulnerabilities is imperative for policymakers and businesses, enabling them to anticipate changes in global trade flows and devise strategies to mitigate potential risks.’*

Reviewer#1’s comment 11: you must explain why you chose US, China, India, Japan and South Korea and the dates 1992-2020.

*Response: Thank you for your constructive feedback. In response, we have included the following explanation in the manuscript:*

*‘We selected U, C, I, J, and K for our study because they are among the most significant players in international trade, contributing extensively to global export dynamics. As such, examining these countries allows for meaningful insights into global trade patterns. Regarding the selected timeline from 1992 to 2020, this range was chosen based on the data availability in the World Integrated Trade Solution (WITS) database. As of May 1, 2023, the earliest available data for the economies we are studying is from 1992, and the most recent data is from 2020. This period allows for a comprehensive, longitudinal study of the changes in trade networks over nearly three decades.’*

Reviewer#1’s comment 12: you need to write the basic theory of analysis.

*Response:* *Thank you for your feedback. We recognize the importance of clearly explaining the foundational theories of our analysis in the Data and Methods section. While we initially detailed our theoretical framework in the introduction, we agree that a brief summary in the methods section could enhance the clarity and cohesion of the manuscript. Therefore, we have updated our Data and Methods section to include a succinct summary of the theoretical underpinnings of each analytical technique we utilized. This encompasses the principles of International Trade Theory informing our examination of trade relationships, the use of Network Analysis in interpreting the structure of trade networks, the application of Time-Series Analysis to identify patterns and trends, and the role of Crisis Theory in understanding disruptions in trade flows. By doing so, we aim to provide clear connections between the theories and their practical application in our analysis, while avoiding redundancy. We trust that this revision adequately addresses your concerns.*

*Below is a summary of the theoretical foundations that inform each analytical method:*

*‘International Trade Theory: This theory undergirds our analysis of trade dynamics among the U.S. (U), China (C), India (I), Japan (J), and South Korea (K). It aids us in interpreting trade imbalances, shifts in trade dominance, and the impact of political and economic crises on these aspects. The principles of this theory guide our examination of these trade relationships over a 29-year period.*

*Network Analysis: This methodological approach is integral to our study as it helps us to unravel the complex structure of international trade relationships. Network Analysis, in our context, enables us to visualize and comprehend the complexities of trade networks, shedding light on their centrality and reciprocity. It allows us to identify dominant players and the characteristics of their trade relationships. Furthermore, we use hierarchical clustering as a method of network theory for community detection.*

*Time-Series Analysis: We employ this analytical method to examine trade data collected at regular intervals. The principles of Time-Series Analysis assist us in identifying underlying trends, cycles, and patterns in the data. This approach is crucial in our investigation of the evolution of the structure of trade networks over time and in assessing the impact of significant events such as political and economic crises.*

*Crisis Theory: Guided by this theory, we delve into how political and economic crises disrupt trade flows and alter the structure of trade networks. It aids us in understanding changes in the centrality and reciprocity of trading partners in response to crises.’*

5.Conclusion

Reviewer#1’s comment 13: policy recommendations are incomplete and inadequate.

*Response: Thank you for your insightful feedback. We added the following sentences in 5.3. Policy recommendations.*

*‘Based on the aforementioned analysis and findings, it seems feasible to propose the following policy recommendations.*

*1. Promotion of Balanced Trade: Given the trend towards more reciprocal network structures, policymakers could consider encouraging practices that nurture this development. This could involve incentives for balanced trading and pursuing trade agreements that uphold equality and mutual benefit. Such measures could promote economic stability and more equitable international relations, though their effects should be closely monitored.*

*2. Adjustment of Trade Focus: The shift of the trade network center from U to C suggests that modifying trade policies and strategies could be beneficial. Actions might include bolstering diplomatic ties, understanding and catering to C's market needs, and encouraging domestic industries to align with C's industrial expansion.*

*3. Strengthening Economic Resilience and Consideration of Global Events: Considering the influence of global events and crises on international trade network structures, policies aimed at enhancing economic resilience and considering geopolitical factors should be contemplated. This could include implementing risk assessment and mitigation measures and diversifying trade partnerships. Incorporating geopolitical risk analysis into trade policy development could help anticipate shifts in trade dynamics, enabling timely and effective responses to evolving global scenarios.*

*These recommendations are proposed with a view to bolstering economic stability, ensuring balanced trade relations, and enhancing resilience against potential shocks. They are based on current findings and should be regularly reassessed in light of new data or changing circumstances.’*

4 Reviewer#1’s comment 14: the comparison of the study with the literature and its original contribution is not given.

*Response: Thank you for your valuable feedback. To address your comment, we have added the following sentences.*

*‘Distinct from the prevailing research, this study introduces a unique methodological approach and offers novel insights. Unlike Garlaschelli and Loffredo's (2004) [18] focus on link reciprocity and the 2015 study's [19] application of multi-regional input-output data, our approach uses the sum of squared trade imbalances to assess network reciprocity and implements clustering analysis to probe trade pattern changes. Unlike the 2022 paper [20] that employed an eigenvector centrality measure, we leverage a time series visualization to explore network structure dynamics. This innovative perspective reveals a divergence in reciprocity trends across product categories, offers insights into shifts in the network center occurring at different times for each product category, and provides evidence of trade network susceptibility to significant crises. Essentially, our work enriches the understanding of global trade networks, offering fresh methodologies and insights into their structure and dynamics.’*

Reviewer #2:

Suggestion: The objective of this manuscript is to analyze the relationship among trade networks of exporting economies in USA, China, Japan and Korea. Data and methods are appropriate. Study has interesting findings. however, I have some concerns about this paper:

*Response: We appreciate your insightful comment and your recognition of the relevance of our data, methods, and the intriguing findings of our research. Your concerns are of utmost importance to us as they aid in refining our manuscript. We will now systematically address each question in sequence.*

Reviewer#2’s Comment 1. The author(s) should clearly present the contribution of this paper to the literature. It should be elaborated on what makes this topic an interesting research area, explaining the novelty of this research output on the subject matter.

*Response: Thank you for your insightful comment regarding the necessity to clearly delineate the contribution of our paper to the existing body of literature. To address this, we have made comprehensive revisions in the introductory section as well as the literature review. Specifically, we have emphasized our novel methodologies, including the use of clustering analysis for time vectors, which stands out as a unique approach in our research.*

*Moreover, our study offers a distinctive perspective on the global trade network by scrutinizing the diverging reciprocity trends across various product categories. This innovative approach has led to the uncovering of novel insights into the structure and dynamics of the global trade network, with a specific focus on its vulnerability to substantial crises.*

*We trust that these modifications will serve to clarify both the novelty and the significance of our research, thereby contributing meaningfully to the progression of understanding within this field.*

Reviewer#2’s Comment 2. The author(s) should clearly explain the empirical approaches implemented in their analysis. The author(s) provide the results of various tests without including a clear explanation of their use and why they were chosen.

*Response2: We apologize for the lack of clarity in the explanation of our empirical approaches. In the revised manuscript, we have provided a more detailed explanation of why we chose our methods and how they are implemented. For example, in 2 Literature review, we added*

*‘The reason for choosing this method (the sum of squared trade imbalances between each pair of actors) is that it allows for the measurement of reciprocity in a way that is simpler and more straightforward than matrix computation methods. ………The reason for choosing this method (hierarchical clustering analysis) is because it was considered highly innovative to use clustering analysis, which is typically used to divide points distributed in space into similar groups, instead for dividing points distributed over time into similar groups in this study. …………The reason for choosing this method (visualization of directed arrows with a standard deviation value 55 or higher in networks) is because visualization has an innate power to appeal to human senses, enabling the measurement of center shift in a manner that is simpler and clearer than sophisticated methods involving eigenvalue computation of matrices.’*

*We hope that these explanations clarify our methodological choices and we welcome any further questions or suggestions on this matter.*

Reviewer#2’s Comment 3. Some arguments should be further analysed to be better supported and the ideas behind them should be developed to help the reader’s understanding (e.g. arguments included a section on the methodology and data used).

*Response 3: We appreciate the reviewer's insightful comments. We acknowledge the validity of these concerns and have made extensive revisions to the manuscript accordingly. In the methodology and data sections, we have provided additional context and background information, further developing the ideas and arguments for our approach. We discuss our choice of data sources in more detail, such as the selection of the WITS database due to its comprehensive and reliable data. We also elaborate on the rationale behind our methodological decisions and discuss the potential limitations of our approach, outlining strategies we have taken to mitigate these limitations. We trust these revisions have addressed the concerns raised, and we welcome any additional feedback.*

Reviewer#2’s Comment 4. Some tables included and the related analysis should be revised as they are not clear for the reader to have a better understanding on the outcome of this empirical analysis.

*Response 4: We appreciate your feedback regarding the clarity of our tables and related analysis. We acknowledge that the presentation of our data could have been clearer in the original manuscript. In response to your comment, we have made several changes to enhance comprehension. Specifically, we have added two tables, one of which presents the data in detail. We have also expanded our discussion and analysis related to these tables to aid the reader in understanding and interpreting our findings. We believe these revisions will help readers to better comprehend the outcomes of our empirical analysis. We welcome any further feedback or suggestions.*

Reviewer#2’s Comment 5. Some conclusions reported cannot be fully supported by the statistical information provided. A careful revision on this matter is needed.

*Response 5: We appreciate this important feedback. In response, we have conducted a thorough review of our conclusions in light of the statistical information provided in our study. We have revised some conclusions to more accurately reflect the data and ensure that all conclusions drawn are well substantiated. Where appropriate, we have also incorporated additional data analysis to strengthen our findings. Furthermore, we have been careful to highlight in the revised manuscript where our findings suggest trends or patterns without definitively establishing causation. Your comment has helped improve the validity and accuracy of our conclusions, and we are grateful for this.*

Reviewer#2’s Comment 6. The quality of English in the paper needs to be improved.

*Response 6: We acknowledge that the quality of English may have affected the readability of the paper and have thoroughly reviewed and revised the manuscript to improve language use and clarity using English editing softwares.*

Reviewer#2’s Comment 7. Please change manuscript title, it seems rough and long.

*Response 7: We greatly appreciate your valuable feedback regarding the title of our manuscript. Upon your suggestion, we have decided to revise the title to:*

*"Dynamics of International Trade: A 30-Year Analysis of Key Exporting Nations."*

Reviewer#2’s Comment 8. Abstract need complete re-writing, please add study problem, method and results.

*Response: We appreciate your feedback. In response, we have completely rewritten the abstract, ensuring to clearly articulate the study problem, method, and results.*
