# Supplementary material for: Dynamics of international Trade: A 30-year analysis of key exporting nations
Source: PLoS One. 2023 Aug 1;18(8):e0289040. doi: 10.1371/journal.pone.0289040 (PMC10393138; doi:10.1371/journal.pone.0289040)
Supplement: Attachment — Submitted filename: Report 2.docx [file pone.0289040.s007.docx]

**REFEREE'S REPORT ON**

**"Shifting Dynamics in International Trade Networks: A Longitudinal Analysis of Major Exporting Economies (1992-2020): A case of the US, China, India, Japan and South Korea"**

**Comments:** The authors studied “shifting dynamics in international trade networks” in US, China, India, Japan and South Korea. This paper needs improvement as has been listed below:

1. **Abstract - All revisions done successfully.**

- The policy proposal of the study's originality and importance should be written in this section.
- Explaining the method and variable in one sentence would be sufficient.
- The policy proposal of the study's originality and importance should be written in this section.

2. **Introduction - All revisions done successfully.**

- The main motivation of the study should be explained correctly in this section.
- The study's importance, purpose and theoretical framework should be discussed in detail.
- Theoretical explanations regarding the main topic is insufficient.
- The general plan of the study should be written at the end of the section.

3. **Literature - All revisions done successfully. Please read and check these studies. Also, please add some current studies to the literature tables.**

[**https://doi.org/10.3390/math10152553**](https://doi.org/10.3390/math10152553)

[**https://doi.org/10.1016/j.jclepro.2023.137517**](https://doi.org/10.1016/j.jclepro.2023.137517)

- It is very important to approach literature studies critically. However, a literature review was not conducted in the study. I suggest creating a literature table.
- The difference of the study from the literature and its contribution to the literature should be explained under this title

4. **Data and Methods** - **All revisions done successfully**

- Analyzes and the findings have not been adequately discussed.
- You must explain why you chose US, China, India, Japan and South Korea and the dates 1992-2020.
- You need to write the basic theory of analysis.

5. **Conclusion - All revisions done successfully**

- Policy recommendations are incomplete and inadequate.
- The comparison of the study with the literature and its original contribution is not given.

**I consider it appropriate to ACCEPT the study for the above-mentioned reasons.**
